# Prevalence and incidence of chronic wounds and related complications: a protocol for a systematic review

**DOI:** 10.1186/s13643-016-0329-y

**Published:** 2016-09-08

**Authors:** Krister Järbrink, Gao Ni, Henrik Sönnergren, Artur Schmidtchen, Caroline Pang, Ram Bajpai, Josip Car

**Affiliations:** 1 Centre for Population Health Sciences (CePHaS), Lee Kong Chian School of Medicine, Nanyang Technological University, 59 Nanyang Drive, Experimental Medicine Building, Singapore, 636921 Singapore; 2 Department of Dermatology, Skåne University Hospital, Lasarettsgatan 15, 221 85 Lund, Sweden; 3 Dermatology and Innate Immunity Laboratory, Lee Kong Chian School of Medicine, Nanyang Technological University, 59 Nanyang Drive, Experimental Medicine Building, Singapore, 636921 Singapore; 4 Medical Library, Lee Kong Chian School of Medicine, 11 Mandalay Road, Singapore, 636921 Singapore; 5 Global eHealth Unit, Department of Primary Care and Public Health, School of Public Health, Imperial College London, 3rd Floor Reynolds Building, St Dunstan’s Road, London, W6 8RP UK

**Keywords:** Chronic wounds, Hard-to-heal ulcers, Wound infection, Ulcer, Wound healing, Diabetic foot, Amputation, Epidemiology, Prevalence, Incidence

## Abstract

**Background:**

Chronic wounds impose a significant and often underappreciated burden to the individual, the healthcare system and the society as a whole. Preliminary literature search suggests that there are at present no reliable estimates on the total prevalence of chronic wounds for different settings and categories of chronic wounds. Such information is essential for policy and planning purposes as the increasing number of elderly and the prevalence of lifestyle diseases point in the direction of an increased burden. Knowledge about the prevalence and incidence of chronic wounds in relation to population characteristics is important for informing healthcare planning and resource allocation. The objective is to present a transparent process for how to review the existing literature on the prevalence and incidence rates of chronic wounds and resulting implications.

**Methods/design:**

We will search electronic bibliographic databases (MEDLINE, EMBASE, the EBM Reviews and Cochrane, Cumulative Index to Nursing and allied Health Literature (CINAHL), PsycINFO, Global Health) and reference lists of included articles. Two investigators will independently screen titles and abstracts and select studies involving adults with chronic wounds. These investigators will also independently extract data using a pre-designed data extraction form that will cover information on demographics, diagnostics including disease prevalence, medical history, hospital and community-based management and outcomes. Subgroup analysis and sensitivity analysis will be performed to address the heterogeneity across studies. Meta-analysis will also be performed if homogeneous group of studies will be found. The collective evidence will be further stratified according to the important background variables if allowed.

**Discussion:**

This study will describe the available epidemiological evidence and summarise prevalence and incidence rates of chronic wounds and related complications. A better understanding of the relationship between population profile and the prevalence of chronic wounds and related complications will be helpful in the development of guidelines for patient management.

**Systematic review registration:**

PROSPERO CRD42016037355

**Electronic supplementary material:**

The online version of this article (doi:10.1186/s13643-016-0329-y) contains supplementary material, which is available to authorized users.

## Background

A chronic wound can be defined as one that has failed to proceed through an orderly and timely reparative process to produce anatomic and functional integrity within a period of 3 months or that has proceeded through the repair process without establishing a sustained, anatomic and functional result [1, 2]. The nomenclature is far from agreed upon, and these wounds are sometimes referred to as hard-to-heal or difficult-to-heal wounds/ulcers, and the time span required for chronicity has been defined in the range 4 weeks up to more than 3 months [2–4]. Based on the causative aetiologies, the Wound Healing Society classifies chronic wounds into four categories: pressure ulcers, diabetic ulcers, venous ulcers and arterial insufficiency ulcers [5]. Chronic wounds are often termed ulcers and can be defined as wounds with a full thickness in depth and a slow healing tendency. Often disguised as a comorbid condition, chronic wounds represent a silent epidemic that affects a large fraction of the world population [6]. It is estimated that 1 to 2 % of the population will experience a chronic wound during their lifetime in developed countries [7]. The dramatic increase in the ageing population will increase these numbers as wound closure is negatively associated with age [8]. Complications of chronic wounds include infection such as cellulitis and infective venous eczema, gangrene, haemorrhage and lower-extremity amputations. Chronic wounds lead to disability and disability worsens wound outcomes resulting in a vicious cycle [9].

Due to the low base rate of complete healing in the natural history [10], chronic wounds have a significant impact on the health and quality of life of patients and their families, causing pain, loss of function and mobility, depression, distress and anxiety, embarrassment and social isolation, financial burden, prolonged hospital stays and chronic morbidity or even death [11]. Evidence suggests that chronic wounds impose significant and often underappreciated burden to the individual, the healthcare system and the society as a whole [12, 13]. In the USA, for example, chronic wounds are reported to affect 6.5 million patients with more than US$25 billion each year spent by the healthcare system on treating wound-related complications [6]. The costly nature of chronic wound management is further confirmed with estimates from the UK, where the cost to the National Health Service of caring for patients with chronic wounds were conservatively estimated at US$3.4–4.6 billion per year (in 2005) representing around 3 % of the total estimated out-turn expenditure on health for the same period [14].

Preliminary literature search suggests that there are at present no reliable estimates on the total prevalence and incidence of chronic wounds for different settings and categories of chronic wounds [15]. The wide disparity of the epidemiological estimates arises mainly from significant heterogeneity in terms of study design and data collection method [15]. The varieties also come from the way of reporting prevalence: some report point prevalence while others report period prevalence [15]. The same disparity is also found in incidence reports. We aim to embrace a larger number of databases and a considerable broader search strategy compared to a previous review [15]. We also intend to thoroughly assess and consider the quality of included studies and carefully pay regard to the applied definitions of chronic wounds. These approaches are believed to provide more reliable estimates on the prevalence for different settings and categories of chronic wounds than previously presented. Such information is essential for policy and planning purposes particularly as the increasing number of elderly and the increase in the prevalence of lifestyle diseases further raise the risk for chronic wounds. The literature review is intended to gather the existing knowledge and identify factors of importance to the prevalence of chronic wounds and to the incidence of complications. The focus is on chronic wounds in the categories of pressure ulcers, diabetic ulcers, venous ulcers and arterial insufficiency ulcers.

Specific review questions are:

- What is the prevalence and incidence of chronic wounds in the categories of pressure ulcers, diabetic ulcers, venous ulcers and arterial insufficiency ulcers, for different settings and subgroups according to internationally published studies?
- What is the prevalence and incidence of infections, gangrene, haemorrhage and limb amputation among patients with chronic wounds in the categories of pressure ulcers, diabetic ulcers, venous ulcers and arterial insufficiency ulcers, for different settings and subgroups according to internationally published studies?

Further knowledge about the distribution of chronic wounds and related complications in different settings and among subgroups is essential for informing healthcare planning and resource allocation. Pooling of such data is also necessary to monitor trends in disease burden and to contribute to the design of further etiological studies.

## Objectives

The overall aim of this systematic review protocol is to present a transparent process for how the information will be collected on the prevalence and incidence of chronic wounds and related complications. This will include the key research questions that this systematic review will address, a description of systematic literature search strategies, criteria for inclusion or exclusion of studies, a description of coding procedures, study quality measures and statistical procedures for the quantitative analysis of data from eligible studies.

## Methods

### Protocol

The methods for this systematic review have been developed according to the recommendations from the Preferred Reporting Items for Systematic Review and Meta-Analysis Protocols (PRISMA-P) 2015 statement [16]. This systematic review protocol has been registered in the International Prospective Register of Systematic reviews (PROSPERO): CRD42016037355). A PRISMA-P file is attached (see Additional file 1).

### Eligibility criteria

#### Population

The population of interest will include adult patients 18 years of age and older with pressure ulcers, diabetic ulcers, venous ulcers and arterial ulcers. Patients with chronic wounds resulting from surgical wounds and skin tumours will be excluded.

#### Outcome

The primary outcome will be the point prevalence, period prevalence, cumulative incidence and incidence rate of chronic wounds in the categories of pressure ulcers, diabetic ulcers, venous ulcers and arterial ulcers, and complications thereof in four categories (infections, gangrene, haemorrhage and limb amputation). Secondary outcomes will be dropping out, the reasons for that, and the number of deaths by cause.

#### Study design

Studies will be restricted by design and observational studies, cross-sectional studies, cohort studies, case-control studies, single arm studies and systemic review/meta-analyses will be included. Non-research letters and editorials, seminar reviews, case studies, case series and animal studies will be excluded. Randomised controlled trials will be excluded based on methodological inappropriateness of research design for the type of questions to be answered.

#### Report characteristics

Searches will be limited to peer-reviewed full text articles in English language, and letters, abstracts and editorials are to be excluded. There will be no geographical limitation on the included studies.

### Information sources

A systematic search of MEDLINE (Ovid), EMBASE (Ovid), EBM Reviews and Cochrane (Ovid), Cumulative Index to Nursing and Allied Health Literature (CINAHL) (EBSCO), PsycINFO (EBSCO) and Global Health (EBSCO) will be undertaken. As we are primarily interested in the contemporary literature on this topic, we will examine publications from January 2000 through December 2015. Contact with authors for further information will be made when necessary. Detailed inclusion and exclusion criteria are presented in Table 1.

**Table 1:** Inclusion and exclusion criteria

	Inclusion criteria	Exclusion criteria
Population	Adult patients (≥18 years of age) with chronic wounds in the categories of pressure ulcers, diabetic ulcers, venous ulcers and arterial insufficiency ulcers and/or complications thereof in terms of infections, gangrene, haemorrhage and limb amputation.	Patients with chronic wounds as a result of surgical wounds and skin tumour.
Study design	Observational studies, cross-sectional studies, cohort studies, case-control studies, single arm studies, systematic review/meta-analyses.	Randomised controlled trials, case studies, case series.
Outcome	Point prevalence, period prevalence, cumulative incidence and incidence rate of chronic wounds in the four categories, and complications thereof.	
Type of publication	Articles with available full text in English language.	Abstract, non-research letters and editorials, seminar reviews.

### Search strategy

We will develop a comprehensive database containing all published studies addressing the prevalence and incidence of chronic wounds (including hard-to-heal wound/ulcers) and complications thereof in general populations. To construct a comprehensive set of possible search terms, we list indexing terms (for example, subject headings and subheadings, publication types) and text words used to describe concept clusters (single words or phrases that may appear in titles or abstracts, both in full and in various truncations). For instance, we search pressure ulcer with its keyword “pressure ulcer” with all heading and subheadings, and then we search alternative keywords “pressure sore” or “bed ulcer” or “bed sore” appearing in the title or abstract in the form of full or truncations. We sought further terms from clinicians and librarians, and from published strategies from other groups. The search strategy was developed by the research team in collaboration with an experienced medical research librarian at the Lee Kong Chian School of Medicine. Additional comments and suggestions were also received from an experienced librarian at the Karolinska Institutet and incorporated. The search was revised, as necessary, and the final MEDLINE search is presented in Additional file 2. After MEDLINE strategy is finalised, it will be adapted to the syntax and subject headings of other databases.

The reference lists of all included articles published from January 2000 and beyond will also be searched for any additional sources of information. An additional search will be made using the newly introduced term “pressure injury” [17] and relevant articles added.

### Study records

#### Data management

We will implement the search strategies and import all references identified to EndNote. The search results from the different electronic databases will be combined in a single EndNote library and we will remove duplicate records of the same reports.

#### Selection process

Two reviewers will independently screen titles and abstracts against eligibility criteria to identify potentially included studies. Specifically, titles and abstracts are included if they indicate that it was a population-based or institutional-based study reporting any relevant information on prevalence or incidence of chronic wounds and/or complications thereof. In the next phase, we will retrieve full-text copies of those articles deemed potentially relevant. Two reviewers will independently assess the full text of the retrieved articles for compliance with our eligibility criteria.

Discrepancies between the two reviewers’ judgement will be resolved by discussion or by the involvement of a third reviewer. Studies, which appeared to be relevant, but excluded at this stage will be listed in the table “Characteristics of excluded studies”, where a reason for exclusion will be noted. Two reviewers will verify the final list of included studies. A PRISMA flow diagram of the study selection procedure will be prepared to provide an overview of the decisions that are made in the data collection process.

#### Data collection process

Two reviewers will independently extract and manage the data for each of the included studies using an electronic data extraction form. We will pilot the data extraction form and amend it according to feedback received from a panel of experienced colleagues. We plan to contact study authors in case of any unclear or missing information. Disagreements between review authors will be resolved by discussion. A third review author will act as an arbiter in case disagreements cannot be resolved. Variations that may depend on age-distribution, the categorisation of ulcer, study design etc. will be described clearly.

### Data items

Data will be extracted on the following.

1. Publication details: title, journal, author, year, city and country, in which the study was conducted, type of publication, and source of funding.
2. Design: type of study (observational studies, cross-sectional, cohort, case-control, single arm studies, systemic review/meta-analyses); aims of study, method of data collection, response rate, recruitment and sampling methods, eligibility (inclusion and exclusion criteria).
3. Study participant details: number of persons interviewed or surveyed, population characteristics including setting, age, gender, ethnicity, demographic information, diagnostics, ulcer specifications (including stage), complications.
4. Data for outcome measures: all reported estimates, or sufficient information to calculate an estimate of the point prevalence, period prevalence, cumulative incidence and incidence rate of chronic wounds in the categories of pressure ulcers, diabetic ulcers, venous ulcers and arterial ulcers, and complications thereof in four categories (infections, gangrene, haemorrhage and limb amputation).
5. Limitations: selection bias, response bias, information bias, limitations of assessment tool(s) used and limitations reported by study authors.

### Risk of bias in individual studies

The risk of bias in included studies will be assessed using a tool developed specifically for conducting quality appraisal of studies in systematic reviews of prevalence data [18]. The instrument addresses critical issues of internal and external validity that must be considered when assessing validity of prevalence data and can be used across different study designs. The instrumental framework evaluates representativeness, recruitment, sample size, reporting, data coverage, condition reliability, statistical analysis and confounding factors using a simple “yes”, “no”, “unclear” or “not/applicable”. The outcome of the assessment will be presented in a table.

Publication bias and selective reporting will be dealt with by critically assessing study findings, plots will be made of outcome variables against sample size [19] and advice will be taken from GRADE guidelines No 5 [20].

### Data synthesis

Relevant data extracted from eligible studies will be presented in evidence tables. Meta-analysis of outcome variables will be conducted if studies are adequately meeting the inclusion criterion and uniform (or clarity) in reporting the outcome estimates. A narrative synthesis will provide summary of the prevalence of chronic wounds and complications thereof according to age, setting and category of wound. These grouping variables will also be used to identify similar patient populations so that quantitative meta-analyses could be made for studies with similar design. The extracted incidence and prevalence figures will be presented with corresponding standard error and 95 % confidence intervals using the exact binomial method as described by Clopper and Pearson [21]. This method produces an exact confidence interval that is directly based on the binomial distribution rather than any approximation to the binomial distribution.

Further, the individual prevalence and incidence rate estimates from observational studies will be pooled using the fixed effect and random effect models [22]. The choice of random-effects model will be based on clinical and methodological diversity across the included studies. We will also perform cumulative meta-analysis, in which estimates for each year will be determined in order to calculate the impact of each added study on the pooled estimates over time. For country-specific pooling of incidence/prevalence data, the Poisson regression technique will be used to adjust the covariates [23]. Where available, the prevalence-adjusted or incidence-adjusted odds ratios relating to the stratification of risk of chronic wound among the patients as discussed in the respective observational studies will also be pooled using random effect models. We will investigate the potential source of heterogeneity related to both methodological and clinical characteristics of the studies which will be assessed by the Cochran’s *Q* test (*P* value <0.05 considered significant) and *I*^2^ (>50 % representing moderate heterogeneity) statistics [24]. In the presence of heterogeneity (*I*^2^ > 25 %) [25], subgroup (e.g. age, sex, setting and clinical categories of chronic wounds) analyses and univariate meta-regression (*P* value <0.10 considered significant given the low power of these tests) will be carried out in order to estimate the effect of study-level covariates on the estimates of incidence and prevalence [26]. Sensitivity analyses will be conducted to identify the study-level factors that best describe the occurrence of chronic wounds. We will also perform influence and outlier analyses to determine the effects of certain studies on the pooled estimates of chronic wound occurrence [27]. These analyses will measure how the estimated parameter of a pooled-analysis would change if noisy studies are eliminated. The effect of these identified outlier studies will be neutralised by excluding them from the random effect model. The publication bias due to study size will also be addressed and adjusted by inverse-variance weighting techniques to provide valid information on the study estimates [26].

## Summary

This systematic review and eventually meta-analysis will be performed to critically examine the world’s relevant literature on the epidemiology of chronic wounds. Specifically, we aim to identify and report the estimated prevalence and incidence of chronic wounds in the categories of pressure ulcers, diabetic ulcers, venous ulcers and arterial insufficiency ulcers and related complications for different settings and subgroups. We will also identify population characteristics associated with chronic wounds in the four categories and with the following complications: infections, gangrene, haemorrhage and limb amputation. Understanding the rates of different categories of complications among patients with chronic wounds could help target patient subgroups that may benefit from early screening, prevention, and treatment efforts. In addition, quantifying the magnitude of chronic wounds will help guide decision-making for the allocation of scarce healthcare resources and funding. A further finding of this systematic review will be the result of the methodological assessment of the published literature. The findings of this review will also be compared with other similar published reviews. Finally, conclusions will be drawn from this systematic review highlighting the prevalence and incidence of chronic wounds, methods of estimation and settings and their correlates. Limitations of the studies will be discussed in detail. Implications of the review as well as suggestions for future research will also be provided.

## Additional files

Additional file 1: PRISMA-P checklist. (DOC 84 kb) Additional file 2: MEDLINE (Ovid) search strategy. (DOCX 32 kb)

## Abbreviations

CINAHL: Cumulative Index to Nursing and allied Health Literature
GRADE: Grading of Recommendations Assessment, Development and Evaluation
PRISMA-P: Preferred Reporting Items for Systematic Reviews and Meta-Analyses Protocols
PROSPERO: International Prospective Register of Systematic Reviews
