# Supplementary material for: Prevalence and incidence of chronic wounds and related complications: a protocol for a systematic review
Source: Syst Rev. 2016 Sep 8;5(1):152. doi: 10.1186/s13643-016-0329-y (PMC5017042; doi:10.1186/s13643-016-0329-y)
Supplement: Additional file 2: — MEDLINE (Ovid) search strategy. (DOCX 32 kb) [file 13643_2016_329_MOESM2_ESM.docx]

**MEDLINE (Ovid) Search Strategy**

1. prevalence.tw. or exp prevalence/

2. incidence.tw. or exp incidence/

3. epidemiology.tw. or exp epidemiology/

4. mortality.tw. or exp mortality/

5. 1 or 2 or 3 or 4

6. exp pressure ulcer/

7. (pressure adj3 ulcer$).tw.

8. (pressure adj3 sore$).tw.

9. exp decubitus ulcer/

10. (decubitus adj3 ulcer$).tw.

11. (decubitus adj3 sore$).tw.

12. (bed adj3 ulcer$).tw.

13. (bed adj3 sore$).tw.

14. bedsore$.tw.

15. 6 or 7 or 8 or 9 or 10 or 11 or 12 or 13 or 14

16. 5 and 15

17. (arterial adj3 ulcer$).tw.

18. (ischemic adj3 ulcer$).tw.

19. (neuropathic adj3 ulcer$).tw.

20. exp Varicose Ulcer/

21. (vascul$ adj3 ulcer$).tw.

22. (varicose adj3 ulcer$).tw.

23. 17 or 18 or 19 or 20 or 21 or 22

24. 5 and 23

25. exp venous ulcer/

26. (venous adj3 ulcer$).tw.

27. exp Stasis ulcer/

28. (stasis adj3 ulcer$).tw.

29. exp Skin ulcer/

30. (skin adj3 ulcer$).tw.

31. 25 or 26 or 27 or 28 or 29 or 30

32. 5 and 31

33. exp Foot Ulcer/

34. (foot adj3 ulcer$).tw.

35. (diabetic adj3 foot).tw.

36. (diabetic adj3 feet).tw.

37. (diabetic adj3 ulcer).tw.

38. exp Leg ulcer/

39. (leg adj3 ulcer$).tw.

40. 33 or 34 or 35 or 36 or 37 or 38 or 39

41. 5 and 40

42. (chronic adj3 wound$).tw.

43. (chronic adj3 sore$).tw.

44. (chronic adj3 ulcer$).tw.

45. (crural adj3 ulcer$).tw.

46. (ulcus adj3 cruris).tw.

47. non-healing wound$.tw.

48. hard to heal.mp.

49. 42 or 43 or 44 or 45 or 46 or 47 or 48

50. 5 and 49

51. (transfemoral amputee$ or transtibial amputee$ or lower limb amputee$ or above-knee amputee$ or below-knee amputee$).tw.

52. exp Amputation/

53. exp Amputation stumps/

54. exp Gangrene/

55. exp soft tissue infections/ or exp wound infection/

56. ((Foot* or feet* or toe* or tissue* or wound*) adj4 (infect* or disease*)).tw.

57. exp Hemorrhage/

58. (h#emorrhag$ or h#morrhag$ or bleed$ or blood loss$).tw.

59. 51 or 52 or 53 or 54 or 55 or 56 or 57 or 58

60. 15 and 59

61. 23 and 59

62. 31 and 59

63. 40 and 59

64. 49 and 59

65. 5 and 60

66. 5 and 61

67. 5 and 62

68. 5 and 63

69. 5 and 64

70. or/65-69

71. 16 or 24 or 32 or 41 or 50

72. 70 or 71

73. limit 72 to (english language and humans and yr="2000 - 2015" and “all adult (19 plus years)”)
